# Assessing the Mental Health Effects of Climate Change: A Narrative Review

**DOI:** 10.1192/j.eurpsy.2025.1189

**Published:** 2025-08-26

**Authors:** H. J. Gomes, M. Pires, C. Freitas, M. Albuquerque, R. M. Freitas

**Affiliations:** 1 Department of Psychiatry and Mental Health, Nordeste Local Health Unit, Bragança; 2 Department of Psychiatry and Mental Health, Guarda Local Health Unit, Guarda; 3 Integrated Responsability Center - Mental Health, Hospital Pedro Hispano, Matosinhos Local Health Unit, Matosinhos, Portugal

## Abstract

**Introduction:**

Climate change refers to any change in climate over time, whether due to natural variability or human activity. It is estimated that between 2030 and 2050, climate change will cause an additional 250,000 deaths annually. Therefore, climate change has emerged as one of the most pressing global challenges of the 21st century, with far-reaching environmental, social, and economic consequences. Beyond its direct physical impacts, growing evidence links climate change to adverse mental health outcomes.

**Objectives:**

This review aims to synthesize current research on the impact of climate change on mental health, identifying key mental health disorders associated with climate-related stressors and highlighting vulnerable populations.

**Methods:**

We performed a narrative literature review by searching PubMed, Google Scholar, and ScienceDirect articles published in English in the last ten years.

**Results:**

Climate change significantly affects mental health, with literature suggesting that for every one-degree Celsius increase in temperature, the incidence of mental health problems rises by approximately 0.9%. Extreme weather events like hurricanes, floods, and wildfires linked to climate change can negatively impact mental health, particularly by contributing to higher rates of depression and post-traumatic stress disorder. Additionally, more gradual shifts in climatic conditions, such as rising temperatures and declining air quality, have also been found to harm mental well-being, contributing to “eco-anxiety” and feelings of helplessness. Vulnerable populations, such as children, the elderly, the mentally ill, and marginalized communities, are at greater risk because of their limited coping resources and increased exposure to the impacts of climate change.

**Conclusions:**

The effects of climate change can be direct or indirect, short-term or long-term, with a growing body of evidence showing its contribution to a wide range of psychological disorders. Policymakers and mental health professionals must consider mental health in climate adaptation strategies and create support systems for affected populations. The European Psychiatric Association (EPA) has recently published a position paper urging the inclusion of mental health considerations in climate strategies. Nevertheless, more research is needed to document the extent of these impacts and the best options for mitigating and treating them.

**Disclosure of Interest:**

None Declared

